# Freezing of Speech Single Questionnaire as a Screening Tool for Cognitive Dysfunction in Patients With Dementia With Lewy Bodies

**DOI:** 10.3389/fnagi.2020.00065

**Published:** 2020-04-28

**Authors:** Pai-Yi Chiu, Guang-Uei Hung, Cheng-Yu Wei, Ray-Chang Tzeng, Ming-Chyi Pai

**Affiliations:** ^1^Department of Neurology, Show Chwan Memorial Hospital, Changhua, Taiwan; ^2^Department of Nuclear Medicine, Chang Bing Show Chwan Memorial Hospital, Changhua, Taiwan; ^3^Department of Neurology, Chang Bing Show Chwan Memorial Hospital, Changhua, Taiwan; ^4^Department of Neurology, Tainan Municipal Hospital, Tainan, Taiwan; ^5^Division of Behavioral Neurology, Department of Neurology, National Cheng Kung University Hospital, College of Medicine, National Cheng Kung University, Tainan, Taiwan; ^6^Alzheimer’s Disease Research Center, National Cheng Kung University Hospital, Tainan, Taiwan; ^7^Institute of Gerontology, College of Medicine, National Cheng Kung University, Tainan, Taiwan

**Keywords:** freezing of speech, fluctuating cognition, dementia with Lewy bodies, Alzheimer’s disease, vascular dementia

## Abstract

**Introduction:**

Freezing phenomenon is a striking feature of Parkinson’s disease. However, it has never been studied in people with dementia with Lewy bodies (DLB). We designed a freezing of speech single questionnaire (FOSSQ) and investigated the frequency and association of freezing of speech (FOS) in patients with DLB and other types of dementia.

**Methods:**

This is a retrospective analysis of data from the project of history-based artificial intelligent computerized dementia diagnostic system. We compared the frequencies of FOS among non-demented (ND) participants, patients with Alzheimer’s disease (AD), vascular dementia (VaD), and DLB. Further, we explored the association factors of FOS in all the participants.

**Results:**

We enrolled 666 individuals with the following disease distribution: 190, ND; 230, AD; 183, VaD; and 63, DLB. Compared to individuals with ND (2.1%), patients with AD (6.1%), or VaD (18.0%), DLB (54.0%) showed a significantly higher frequency of positive FOS (all *p* < 0.001). The association factors of FOS were older age, more severe dementia, more severe motor dysfunction, fluctuating cognition, visual hallucinations, parkinsonism, rapid eye movement sleep behavior disorder, attention, mental manipulation, and language.

**Conclusion:**

Our study showed that the informant-based FOSSQ may be a practical screening tool for discriminating DLB from individuals with ND or other forms of dementia. The FOSSQ can be applied in clinical practice as well as on the artificial intelligent platform.

## Introduction

Freezing phenomenon is a striking feature of Parkinson’s disease (PD). Freezing of gait (FOG) is a form of akinesia and is one of the most disabling symptoms of PD (Giladi et al., 2001). Previous studies on FOG in PD have reported a prevalence of 7.1–46% (Giladi et al., 2001; Macht et al., 2007) according to different criteria. However, there have only been a few studies on the freezing of speech (FOS) phenomenon (Ackermann et al., 1993; Giladi et al., 1997; Louis et al., 2001; Park et al., 2014; Vercruysse et al., 2014) in PD. Further, freezing phenomenon has rarely been mentioned or studied in neurological disorders other than PD, with some mentioning the phenomenon of FOS sharing a similar neural mechanism with FOG (Park et al., 2014; Vercruysse et al., 2014). The underlying mechanism of the freezing phenomenon is probably related to dysfunction of the fronto-striatal circuits (Vercruysse et al., 2014). To our knowledge, there have been no studies on FOS in dementia with Lewy bodies (DLB) or other degenerative types of dementia. DLB is one of the Lewy body diseases (LBD) and it shares a similar pathological manifestation with PD, especially PD with dementia (PDD) (Kosaka et al., 1984; Jellinger, 2018). Therefore, patients with DLB should demonstrate similar freezing phenomenon as PD/PDD. It remains unclear whether FOS is a deficit of language or other cognitive functions. However, language dysfunction is indeed one of the common cognitive features of a diagnosis of dementia due to Alzheimer’s disease (AD) or other forms of dementia (McKeith et al., 2005; McKhann et al., 2011; Hardy et al., 2016). Similarly, the association factors of FOS have not yet been well studied.

To study FOS, we designed a simple informant-based FOS single questionnaire (FOSSQ) that was embedded in the History-based Artificial Intelligent Clinical Dementia Diagnostic System (HAICDDS) (Lin et al., 2018; Chiu et al., 2019). We aimed to investigate the frequency and association of FOS in DLB and other types of dementia. We proposed that since the FOS phenomenon is a striking feature of PD, it could be an important feature of DLB because both language (Lin et al., 2018) and motor deficits (McKeith et al., 2005) are common clinical presentations of DLB. Based on the findings that the underlying mechanism of the freezing phenomenon is probably related to dysfunction of the fronto-striatal circuits (Vercruysse et al., 2014), we proposed that FOS could be a deficit involving language and motor dysfunction as well as dysfunction of other cognitive functions such as attention and executive dysfunction. Similarly, as a striking clinical phenomenon, we also proposed that FOS may involve fluctuation of attention and cognition, and thus performed fluctuation scale comparisons.

## Materials and Methods

This is a sub-study of the project of HAICDDS, which is currently used as a registration platform in the Show Chwan Healthcare System (Lin et al., 2018; Chiu et al., 2019). Participants who visited either hospital of the healthcare system with suspected cognitive or motor dysfunction were registered with their demographical, clinical, cognitive, neuropsychiatric, motor, laboratory, and neuroimaging data in the database. The HAICDDS questionnaire is part of the database for recording of clinical history. It is composed of 100 questions designed after a consensus meeting of 12 neurologists, one geriatric psychiatrist, three nuclear medicine doctors, and one neuroradiologist. The fundamental structure of the questionnaire is similar to that of history-taking used by physicians for acquiring detailed clinical information. Before commencing the project, thirty patients with their informants were assessed by neuropsychologists from three centers and the reproducibility was studied using the interrater reliability analysis. Cronbach’s alpha coefficient was calculated to estimate the reliability of the entire novel screening questionnaire. The original writing of FOSSQ was in Chinese as follows: 跟人講話時常常思緒突然中斷，腦袋好像一片空白，難以互動嗎 (The tentative translation to English is: “When speaking, does he/she pause frequently, seem blank, and have trouble communicating?”). In this sub-study, we analyzed and compared the FOSSQ results among non-demented (ND) participants as well as participants with AD, vascular dementia (VaD), and DLB. Further, we studied the association factors of FOS among the participants.

### Diagnosis of AD, VaD, or DLB

A diagnosis of AD was made according to the criteria for dementia due to AD developed by the National Institute on Aging-Alzheimer’s Association workgroups on diagnostic guidelines for AD (McKhann et al., 2011). A diagnosis of VaD was made according to the criteria for probable VaD or possible VaD in the 2011 AHA/ASA criteria for vascular cognitive impairment (VCI) (Gorelick et al., 2011). A diagnosis of DLB was made according to the revised consensus criteria for probable DLB developed by the fourth report of the DLB consortium (McKeith et al., 2017).

### Diagnosis of ND, or Different Stages of Dementia

ND participant was diagnosed with a global CDR (Morris, 1993) score of 0 or 0.5 without significantly impaired activities of daily living, which was defined by an Instrumental Activities of Daily Living (IADL) score greater than 6 (Mao et al., 2018). A diagnosis of dementia was made according to the criteria for dementia developed by the NIA-AA (McKhann et al., 2011). Specifically, people with dementia had impairments in two cognitive domains or more as well as declined daily functions (at least one of the domains of community affairs, home hobbies, and personal care with a CDR score ≥ 0.5 and an IADL score ≤ 6) (Mao et al., 2018). Dementia severity was defined by the global CDR scale score; specifically, participants with a global CDR score of 0.5, 1, 2, and 3 were defined as having very mild, mild, moderate, and severe dementia, respectively (Morris, 1993).

### Procedure of the Study

This is a retrospective analysis of data from the HAICDDS, which is currently applied in three centers in Taiwan (two in central Taiwan and one in southern Taiwan). Daily function was assessed using the Instrumental Activities of Daily Living (IADL) Scale (Lawton and Brody, 1969). Cognitive function was assessed using the Cognitive Abilities Screening Instrument (CASI) (Lin et al., 2002) and the Montreal Cognitive Assessment (MoCA) (Chen et al., 2016). Neuropsychiatric symptoms were assessed using the Neuropsychiatric Inventory (NPI) (Cummings, 1988). Language function was assessed using the language subscales in the CASI and HAICDDS (HAICDDS-Language) (Lin et al., 2018). Language domain in CASI screens cognitive performance on reading, writing, naming, and comprehension whereas, language questions in HAICDDS acquire information on speech fluency, comprehension, naming, volume, and tone based on clinical history (Lin et al., 2018). Trained neuropsychologists administered the cognitive tests and NPI to all the patients.

### Statistics

The Chinese version of SPSS 22.0 for Windows (IBM, SPSS Inc., Chicago) was used for statistical analyses. Between-group comparisons of demographic data, neuropsychological tests, CDR sum of boxes (CDR-SB), IADL score, MoCA score, CASI score, NPI total score (NPI-sum), motor subscale of the Unified Parkinson’s Disease Rating Scale (UPDRS-M), and DLB features were analyzed using independent t-test or one-way ANOVA with either Bonferroni or Dunnett T3 *post hoc* analysis according to the homogeneity of variance. Between-group comparisons of demographic and background characteristics with positive FOSSQ (FOSSQ +) and negative FOSSQ (FOSSQ-) were adjusted for age and disease severity. Comparisons of each cognitive domain in the CASI between FOSSQ + and FOSSQ- groups were adjusted for age, gender, and disease severity. Comparison of frequency of symptom fluctuation in the Mayo Fluctuation Composite Score (MFCS) and language symptoms in the HAICDDS-Language between FOSSQ + and FOSSQ- groups were adjusted for age and disease severity.

### Ethical Consideration

The participants were selected from a registry-based database of the Show Chwan Health System. The study design was retrospective, and the data were anonymously analyzed. The Committee for Medical Research Ethics of Show Chwan Memorial Hospital reviewed the project and the Data Inspectorate approved the study.

## Results

We enrolled 666 individuals with the following disease distribution: 190, ND; 230, AD; 183, VaD; and 63, DLB. The frequency of FOSSQ + in patients with CDR scores of 0, 0.5, 1, 2, and 3 were 3.8, 3.0, 15.2, 26.1, and 43.5%, respectively. Compared to patients with ND (2.1%), AD (6.1%), or VaD (18.0%), those with DLB (54.0%) showed a significantly higher frequency of FOSSQ + (all *p* < 0.001). Comparison of demographic and background characteristics among patients with ND, AD, VaD, and DLB revealed significant differences in all the parameters (*p* < 0.001), except for informant age or education (Table 1).

**TABLE 1:** Comparison of demographic data among ND, AD, VaD, and DLB groups.

Groups	ND	AD	VaD	DLB	F/x^2^	*p*
N	190	230	183	63		
Age, year	68.1 ± 11.4	80.1 ± 8.6	73.7 ± 10.7	80.8 ± 7.0	57.24	<0.001
Female, N (%)	100 (52.6)	154 (67.0)	77 (42.1)	36 (57.1)	29.19	<0.001
Education, year	7.4 ± 4.9	4.4 ± 4.5	6.0 ± 4.8	5.3 ± 13.0	9.15	<0.001
CDR, 0/0.5/1/2/3	53/137/0/0/0	0/85/77/51/17	0/66/52/48/17	0/9/22/20/12	324.18	<0.001
CDR-SB	0.7 ± 0.7	6.7 ± 4.4	7.2 ± 5.0	9.7 ± 5.2	128.61	<0.001
IADL	7.7 ± 0.6	2.7 ± 2.7	2.6 ± 2.9	1.4 ± 2.3	235.16	<0.001
MoCA	20.0 ± 6.0	7.4 ± 5.5	9.1 ± 6.8	7.1 ± 6.5	175.02	<0.001
CASI	81.5 ± 12.1	44.8 ± 21.5	47.8 ± 24.6	43.2 ± 23.3	139.26	<0.001
NPI-sum	3.4 ± 5.0	5.2 ± 6.9	8.3 ± 9.3	15.6 ± 14.0	31.02	<0.001
UPDRS-M	7.1 ± 7.1	11.4 ± 12.0	30.6 ± 14.6	28.9 ± 11.1	37.76	<0.001
**DLB features, N (%)**						
Fluctuation	4 (2.1)	32 (13.9)	48 (26.2)	32 (50.8)	91.61	<0.001
VH	1 (0.5)	10 (4.3)	16 (8.7)	29 (46.0)	136.07	<0.001
Parkinsonism	32 (16.8)	42 (18.3)	73 (39.9)	59 (93.7)	157.39	<0.001
RBD	17 (8.9)	13 (5.7)	13 (7.1)	34 (54.0)	123.49	<0.001
DaTabN	2 (6.9)	3 (20.0)	8 (47.1)	19 (79.2)	31.94	<0.001
**Informant**						
Age, year	58.8 ± 13.3	54.9 ± 14.3	55.7 ± 13.7	54.3 ± 13.7	1.96	0.120
Education, year	11.1 ± 7.5	11.1 ± 4.3	10.2 ± 4.8	11.1 ± 4.9	0.60	0.613

*N, Number of participants; ND, Non-demented; AD, Alzheimer disease; VaD, vascular dementia; DLB, Dementia with Lewy bodies; CDR, Clinical Dementia Rating; IADL, Instrumental Activities of Daily Living; MoCA, Montreal Cognitive Assessment; CASI, Cognitive Abilities Screening Instrument; NPI-sum, The sum of score of Neuropsychiatric Inventory; UPDRS-M, motor subscale of the Unified Parkinson’s Disease Rating Scale in 28 NC, 34 AD, 34 VCI, and 25 DLB; VH, visual hallucinations; RBD, REM sleep behavior disorder; DaTabN, abnormal dopamine transporter imaging in 29 NC, 15 AD, 17 VCI, and 24 DLB.*

Comparison of demographic and background characteristics between the FOSSQ + and FOSSQ- groups before adjustment revealed that the FOSSQ + group had a significantly older age and more severe dementia stages. Further, there were significant differences in all other parameters (*p* < 0.001) except for gender, education, and history of cerebrovascular accident (Table 2). After adjustment for age and disease severity, FOSSQ + had more males and higher DLB features including UPDRS-M, cognitive fluctuations, visual hallucinations, parkinsonism, and REM sleep behavior disorder (Table 2).

**TABLE 2:** Comparison of demographic and background characteristics between groups with FOSSQ + and FOSSQ – adjusted for age and disease severity according to CDR.

	Mean ± SD		Non-adjusted		Adjusted	
	FOSSQ +	FOSSQ−	t/χ^2^	*p*	OR (95% CI)	*p*
N	85	581				
Age, years	77.3 ± 10.1	74.6 ± 11.3	2.20	0.028	NA	
CDR, 0/0.5/1/2/3	2/9/23/31/20	51/288/128/88/26	87.79	<0.001	NA	
Female, N (%)	39 (45.9)	328 (56.3)	3.35	NS	0.56 (0.34–0.92)	0.023
Education, years	5.7 ± 11.3	5.8 ± 4.9	−0.14	NS	1.01 (0.98–1.05)	NS
IADL	1.4 ± 2.4	4.3 ± 3.3	−7.97	<0.001	0.95 (0.77–1.16)	NS
MoCA	6.3 ± 6.2	12.2 ± 8.2	−6.36	<0.001	0.98 (0.92–1.04)	NS
CASI	37.7 ± 23.6	58.6 ± 25.4	−7.18	<0.001	0.99 (0.98–1.01)	NS
NPI	14.0 ± 11.8	5.4 ± 7.9	8.73	<0.001	1.05 (1.03–1.08)	<0.001
UPDRS-M	30.9 ± 19.9	18.2 ± 16.6	8.34	<0.001	1.02 (1.01–1.03)	0.002
**DLB features**						
Fluctuation	56 (65.9)	60 (10.3)	159.11	<0.001	10.22 (5.76–18.13)	<0.001
VH	26 (30.6)	30 (5.2)	62.24	<0.001	4.18 (2.20–7.95)	<0.001
Parkinsonism	58 (68.2)	148 (25.5)	63.27	<0.001	4.68 (2.77–7.91)	<0.001
RBD	21 (24.7)	56 (9.6)	16.46	<0.001	2.84 (1.52–5.29)	<0.001
DaTabN	12 (63.2)	20 (30.3)	6.78	0.009	1.16 (0.23–3.29)	NS
History of CVA	26 (30.6)	128 (22.0)	3.06	NS	1.11 (0.63–1.94)	NS

*FOSSQ, Freezing of Speech Single Questionnaire; CDR, Clinical Dementia Rating scale; N, number of cases; NA, not applicable; NS, not significant; OR, odds ratio; IADL, Instrumental Activities of Daily Living; MoCA, Montreal Cognitive Assessment; CASI, Cognitive Abilities Screening Instrument; NPI, total score of the twelve-domain Neuropsychiatric Inventory; UPDRS-M, motor subscale of the Unified Parkinson’s Disease Rating Scale in 28 NC, 34 AD, 34 VCI, and 25 DLB; VH, visual hallucinations; RBD, REM sleep behavior disorder; DaTabN, abnormal dopamine transporter imaging in 29 NC, 15 AD, 17 VCI, and 24 DLB; CVA, cerebrovascular accident.*

Comparison of each cognitive domain in the CASI between the FOSSQ + and FOSSQ- groups before adjustment revealed that the FOSSQ + group had significantly lower scores in all domains (*p* < 0.001). After adjustment for age and disease severity, the FOSSQ + group had significantly lower scores only in the attention and language domains (Table 3).

**TABLE 3:** Comparison of each cognitive domain in CASI between groups with FOSSQ + and FOSSQ – adjusted for age, education, gender, and disease severity according to CDR.

	Mean ± SD		Non-adjusted		Adjusted	
	FOSSQ +	FOSSQ−	t	*p*	OR (95% CI)	*p*
N	85	581				
RMM	6.2 ± 3.3	8.1 ± 2.7	−5.26	<0.001	0.98 (0.88–1.09)	NS
RCM	3.6 ± 3.2	5.9 ± 4.1	−4.90	<0.001	1.01 (0.92–1.11)	NS
ATT	2.3 ± 2.1	5.0 ± 2.3	−10.00	<0.001	0.67 (0.59–0.77)	<0.001
MEN	1.6 ± 2.6	4.0 ± 3.5	−5.99	<0.001	0.85 (0.75–0.95)	0.006
ORI	7.4 ± 5.6	11.1 ± 5.9	−5.54	<0.001	1.03 (0.96–1.10)	NS
ABS	2.8 ± 2.3	4.3 ± 2.4	−5.47	<0.001	0.93 (0.86–1.15)	NS
LAN	5.8 ± 3.0	7.9 ± 2.2	−7.52	<0.001	0.89 (0.80–0.99)	0.044
DRAW	4.5 ± 3.8	6.6 ± 4.0	−4.60	<0.001	1.02 (0.94–1.10)	NS
ANM	3.6 ± 3.2	5.8 ± 3.5	−5.58	<0.001	1.04 (0.93–1.15)	NS

*CASI, Cognitive Abilities Screening Instrument; FOSSQ, Freezing of Speech Single Questionnaire; CDR, Clinical Dementia Rating scale; N, number of cases; NA, not applicable; NS, not significant; OR, odds ratio; RMM, remote memory; RCM, recent memory; ATT, attention; MEN, mental manipulation; ORI, orientation; ABS, abstract thinking; LAN, language; DRAW, drawing; ANM, animal naming.*

Comparison of frequency of symptom fluctuation in the MFCS and language symptoms in the HAICDDS-Language between FOSSQ + and FOSSQ- before and after adjustment for age and disease severity showed that FOSSQ + had a significantly higher frequency in all domains in the MFCS and HAICDDS-Language except for language impairment, which occurred much earlier than the other symptoms (HAICDDS-L7) (Table 4). Given our recent finding that HAICDDS-L8 has the highest power for discrimination of DLB when compared to other types of dementia (Lin et al., 2018), we further combined FOSSQ with HAICDDS-L8 and found that the positive rate for each question were DLB (61.9%), VaD (29.0%), AD (7.8%), and ND (2.6%).

**TABLE 4:** Comparison of frequency of fluctuation symptoms in MFCS and language symptoms in HAICDDS-Language between groups with FOSSQ + and FOSSQ – adjusted for age and disease severity according to CDR.

	Mean ± SD		Non-adjusted		Adjusted	
	FOSSQ +	FOSSQ −	t	*p*	OR (95% CI)	*p*
N	85	581				
**MFCS**						
Disorganized speech	37 (43.5)	34 (5.9)	110.52	<0.001	5.74 (3.17–10.41)	<0.001
Drowsy/lethargic	57 (67.1)	156 (26.9)	55.11	<0.001	3.39 (1.99–5.77)	<0.001
Sleep > 2 h	63 (74.1)	121 (20.8)	105.32	<0.001	6.68 (3.77–11.82)	<0.001
Staring into space	57 (67.1)	73 (12.6)	140.17	<0.001	7.88 (4.41–14.08)	<0.001
MFCS total score	2.5 ± 1.2	0.7 ± 1.0	15.69	<0.001	3.12 (2.38–4.10)	<0.001
**HAICDDS-Language**						
L1	60 (70.6)	173 (29.8)	87.76	<0.001	3.09 (1.78–5.37)	<0.001
L2	68 (80.0)	199 (34.3)	96.55	<0.001	3.01 (1.54–5.90)	0.001
L3	54 (63.5)	140 (24.1)	91.10	<0.001	2.38 (1.35–4.19)	0.003
L4	21 (24.7)	22 (3.8)	60.31	<0.001	4.70 (2.37–9.56)	<0.001
L5	59 (69.4)	155 (26.7)	62.10	<0.001	2.65 (1.48–4.77)	0.001
L6	61 (71.8)	198 (34.1)	74.68	<0.001	2.87 (1.66–4.96)	<0.001
L7	2 (2.4)	6 (1.0)	0.89	NS	3.33 (0.56–19.64)	NS
L8	28 (32.9)	30 (5.2)	71.97	<0.001	4.96 (2.62–9.38)	<0.001
Language total score	6.0 (3.2)	1.9 (2.4)	13.57	<0.001	1.45 (1.30–1.61)	<0.001

*MFCS, Mayo Fluctuation Composite Score; HAICDDS-Language; Language subscale in the History-based Artificial Intelligent Clinical Dementia Diagnostic System; FOSSQ, Freezing of Speech Single Questionnaire; CDR, Clinical Dementia Rating scale; N, number of cases; NA, not applicable; NS, not significant; OR, odds ratio; L1, Does his/her speaking become noticeably less fluent? L2, Can’t he/she understand words or sentences of others? L3, Is it difficult to express a sentence completely? L4, Does he/she repeat the same words or repeat the words of others? L5, Does he/she speak very little? L6, Does he/she have trouble finding words or names? L7, Does language impairment occur early and much earlier than other symptoms? L8, Does his/her speech reduce pitch range (monotone) and reduced volume (hypophonia)? Any, at least one scale of the language questionnaire was positive.*

## Discussion

As a sub-study of the project HAICDDS, we analyzed data from a relatively large population (666 individuals) and obtained important results. First, we successfully demonstrated that even a single question could provide good discrimination among dementia stages and subtypes. The frequencies of FOSSQ + in patients with CDR scores of 0, 0.5, 1, 2, and 3 were 3.8, 3.0, 15.2, 26.1, and 43.5%, respectively, indicating that the frequency of FOS significantly increased with increase in dementia severity. Regarding discrimination of dementia subtypes, the FOS phenomenon was found more often in patients with DLB (54.0%) compared to those with either AD (6.1%) or VaD (18.0%). The frequency of FOSSQ + was lower among females, which is consistent with previous findings on freezing phenomenon in Parkinson’s disease (Park et al., 2014).

Second, the frequency of FOS was not only highest in patients with DLB but was also more significantly associated with DLB features (*p* < 0.001), including cognitive fluctuations (OR = 10.22), visual hallucinations (OR = 4.18), parkinsonism (OR = 4.68), and REM sleep behavior disorder (OR = 2.84). Motor dysfunction according to the UPDRS-M is more severe in the FOSSQ + group (OR = 1.02; *p* = 0.002). Therefore, it is reasonable to consider FOS as part of the characteristic features in the diagnosis of DLB than that of ND or other types of dementia. The dopamine transporter imaging is not significantly different after adjustment, however. We proposed that non-DLB participants who performed dopamine transporter imaging were comorbid with motor dysfunction and were highly suspected to have parkinsonism. Hence, a relatively high percentage of DaTabN was found among these patients (47.1 and 20% in VaD and AD). Our next piece of research to address this issue and to clarify the association of FOS and dopamine transporter uptake in the striatal area is underway.

Third, after adjustment for age and dementia severity, the FOSSQ + demonstrated a strong relationship with dysfunctions in attention, mental manipulation, and language domains in the CASI. This indicated that FOS might be a combination of these types of cognitive dysfunction, which is consistent with our hypothesis. Some researchers may argue that using the cognitive screening tool CASI, attention domain screens only registration and repetition and language domain screening cognitive performance on reading, writing, naming, and comprehension. Whether attention and language subscales in CASI represent the actual functions of attention and language might be controversial based only on a cognitive screening tool. Further detailed and specific neuropsychological tests to clarify the association of FOS with cognitive functions will be necessary.

Fourth, it is consistent with our recent findings of language dysfunction being more severe in patients with DLB than those with other dementia using the HAICDDS-Language questionnaire (Lin et al., 2018). In the study, we found that the discriminative ability of the HAICDDS-Language, especially the HAICDDS-L8 (“Does his/her speech reduce pitch range and reduced volume?”) among patients with DLB/PDD and AD was robust (OR = 9.16; 95% CI: 6.33–13.25) (Lin et al., 2018). Therefore, in the current study, we combined the FOSSQ with HAICDDS-L8 and found an increase of positive rate in DLB (61.9%) which is much higher than those in VaD (29.0%), AD (7.8%), or ND (2.6%).

Finally, in the HAICDDS project, we have continuously provided evidence that the combination of multiple standardized and structured questions is more powerful and can be optimized using artificial intelligence with machine learning techniques. This was the original idea behind the design of the HAICDDS questionnaire.

This study has several limitations. First, we used a single questionnaire for detecting FOS in patients with DLB and other forms of dementia; therefore, further studies using more tools are required to confirm the findings. Second, we used the original Taiwanese version of the questionnaire, which, although we translated to English, would call for a more precise and colloquial translation. Third, our study was conducted in only three centers in Taiwan. Therefore, the findings on the prevalence and association factors of FOS are not generalizable to all patients with DLB or other types of dementia.

## Conclusion

In conclusion, our study showed that the informant-based single questionnaire FOSSQ can be a practical screening tool for the discrimination of DLB from ND or other types of dementia. The FOSSQ can be applied in clinical practice as well as in the dementia registration platform. Further machine learning techniques using artificial intelligence could improve the accuracy and efficiency of the questionnaire.

## Data Availability Statement

The raw data supporting the conclusions of this article will be made available by the authors, without undue reservation, to any qualified researcher.

## Ethics Statement

The studies involving human participants were reviewed and approved by the Show Chwan Memorial Hospital. Written informed consent for participation was not required for this study in accordance with the national legislation and the institutional requirements.

## Author Contributions

P-YC undertook the literature search and data analysis, edited the Author Contributions, and was mainly responsible for revisions and drafts of the manuscript. M-CP participated in the data analysis and contributed to revisions and the final draft of the manuscript. G-UH undertook the literature search and contributed to revisions. C-YW contributed to revisions of the manuscript. R-CT undertook the literature search and contributed to revisions.

## Conflict of Interest

P-YC work has been partly supported by the Show Chwan Memorial Hospital. The remaining authors declare that the research was conducted in the absence of any commercial or financial relationships that could be construed as a potential conflict of interest.

## References

[B1] Ackermann H. Grone B. F. Hoch G. Schonle P. W. (1993). Speech freezing in Parkinson’s disease: a kinematic analysis of orofacial movements by means of electromagnetic articulography. *Folia Phoniatr.* 45 84–89. 10.1159/000266222 8325574

[B2] Chen K. L. Xu Y. Chu A. Q. Ding D. Liang X. N. Nasreddine Z. S. (2016). Validation of the Chinese version of montreal cognitive assessment basic for screening mild cognitive impairment. *J. Am. Geriatr. Soc.* 64 e285–e290. 10.1111/jgs.14530 27996103

[B3] Chiu P. Y. Tang H. Wei C. Y. Zhang C. Hung G. U. Zhou W. (2019). NMD-12: a new machine-learning derived screening instrument to detect mild cognitive impairment and dementia. *PLoS One* 14:e0213430. 10.1371/journal.pone.0213430 30849106 PMC6407752

[B4] Cummings J. L. (1988). Intellectual impairment in Parkinson’s disease: clinical, pathologic, and biochemical correlates. *J. Geriatr. Psychiatry Neurol.* 1 24–36. 10.1177/089198878800100106 2908099

[B5] Giladi N. Kao R. Fahn S. (1997). Freezing phenomenon in patients with parkinsonian syndromes. *Mov. Disord.* 12 302–305. 10.1002/mds.870120307 9159723

[B6] Giladi N. McDermott M. P. Fahn S. Przedborski S. Jankovic J. Stern M. (2001). Freezing of gait in PD: prospective assessment in the DATATOP cohort. *Neurology* 56 1712–1721. 10.1212/wnl.56.12.1712 11425939

[B7] Gorelick P. B. Scuteri A. Black S. E. Decarli C. Greenberg S. M. Iadecola C. (2011). Vascular contributions to cognitive impairment and dementia: a statement for healthcare professionals from the american heart association/american stroke association. *Stroke* 42 2672–2713. 10.1161/STR.0b013e3182299496 21778438 PMC3778669

[B8] Hardy C. J. Buckley A. H. Downey L. E. Lehmann M. Zimmerer V. C. Varley R. A. (2016). The language profile of behavioral variant frontotemporal dementia. *J. Alzheimers. Dis.* 50 359–371. 10.3233/jad-150806 26682693 PMC4740928

[B9] Jellinger K. A. (2018). Dementia with Lewy bodies and Parkinson’s disease-dementia: current concepts and controversies. *J Neural Transm.* 125 615–650. 10.1007/s00702-017-1821-1829 29222591

[B10] Kosaka K. Yoshimura M. Ikeda K. Budka H. (1984). Diffuse type of lewy body disease: progressive dementia with abundant cortical Lewy bodies and senile changes of varying degree–a new disease? *Clin. Neuropathol.* 3 185–192. 6094067

[B11] Lawton M. P. Brody E. M. (1969). Assessment of older people: self-maintaining and instrumental activities of daily living. *Gerontologist* 9 179–186. 10.1093/geront/9.3_Part_1.179 5349366

[B12] Lin C. M. Hung G. U. Wei C. Y. Tzeng R. C. Chiu P. Y. (2018). An informant-based simple questionnaire for language assessment in neurodegenerative disorders. *Dement. Geriatr. Cogn. Disord.* 46 207–216. 10.1159/000493540 30336484

[B13] Lin K. N. Wang P. N. Liu C. Y. Chen W. T. Lee Y. C. Liu H. C. (2002). Cutoff scores of the cognitive abilities screening instrument. Chinese version in screening of dementia. *Dement. Geriatr. Cogn. Disord.* 14 176–182. 10.1159/000066024 12411759

[B14] Louis E. D. Winfield L. Fahn S. Ford B. (2001). Speech dysfluency exacerbated by levodopa in Parkinson’s disease. *Mov. Disord.* 16 562–565. 10.1002/mds.1081 11391759

[B15] Macht M. Kaussner Y. Moller J. C. Stiasny-Kolster K. Eggert K. M. Kruger H. P. (2007). Predictors of freezing in Parkinson’s disease: a survey of 6,620 patients. *Mov. Disord.* 22 953–956. 10.1002/mds.21458 17377927

[B16] Mao H. F. Chang L. H. Tsai A. Y. Huang W. W. Tang L. Y. Lee H. J. (2018). Diagnostic accuracy of instrumental activities of daily living for dementia in community-dwelling older adults. *Age Ageing* 47 551–557. 10.1093/ageing/afy021 29528375

[B17] McKeith I. G. Boeve B. F. Dickson D. W. Halliday G. Taylor J. P. Weintraub D. (2017). Diagnosis and management of dementia with Lewy bodies: fourth consensus report of the DLB Consortium. *Neurology* 89 88–100. 10.1212/wnl.0000000000004058 28592453 PMC5496518

[B18] McKeith I. G. Dickson D. W. Lowe J. Emre M. O’Brien J. T. Feldman H. (2005). Diagnosis and management of dementia with lewy bodies: third report of the DLB consortium. *Neurology* 65 1863–1872. 10.1212/01.wnl.0000187889.17253.b1 16237129

[B19] McKhann G. M. Knopman D. S. Chertkow H. Hyman B. T. Jack CR Jr Kawas C. H. (2011). The diagnosis of dementia due to Alzheimer’s disease: recommendations from the National Institute on Aging-Alzheimer’s association workgroups on diagnostic guidelines for Alzheimer’s disease. *Alzheimer’s Dement.* 7 263–269. 10.1016/j.jalz.2011.03.005 21514250 PMC3312024

[B20] Morris J. C. (1993). The clinical dementia rating (CDR): current version and scoring rules. *Neurology* 43 2412–2414. 10.1212/wnl.43.11.2412-a 8232972

[B21] Park H. K. Yoo J. Y. Kwon M. Lee J. H. Lee S. J. Kim S. R. (2014). Gait freezing and speech disturbance in Parkinson’s disease. *Neurol. Sci.* 35 357–363. 10.1007/s10072-013-1519-1511 23975521

[B22] Vercruysse S. Gilat M. Shine J. M. Heremans E. Lewis S. Nieuwboer A. (2014). Freezing beyond gait in Parkinson’s disease: a review of current neurobehavioral evidence. *Neurosci. Biobehav. Rev.* 43 213–227. 10.1016/j.neubiorev.2014.04.010 24769288

